# Gas Chromatography–Mass Spectroscopy-Based Metabolomics Analysis Reveals Potential Biochemical Markers for Diagnosis of Gestational Diabetes Mellitus

**DOI:** 10.3389/fphar.2021.770240

**Published:** 2021-11-19

**Authors:** Beata A. Raczkowska, Patrycja Mojsak, David Rojo, Beata Telejko, Magdalena Paczkowska–Abdulsalam, Justyna Hryniewicka, Anna Zielinska–Maciulewska, Malgorzata Szelachowska, Maria Gorska, Coral Barbas, Adam Kretowski, Michal Ciborowski

**Affiliations:** ^1^ Clinical Research Centre, Medical University of Bialystok, Bialystok, Poland; ^2^ Centro de Metabolómica y Bioanálisis (CEMBIO), Facultad de Farmacia, Universidad CEU San Pablo, Campus Montepríncipe, Madrid, Spain; ^3^ Department of Endocrinology, Diabetology and Internal Medicine, Medical University of Bialystok, Bialystok, Poland

**Keywords:** gestational diabetes mellitus, biomarkers, metabolomics, serum, quantitative analysis, gas chromatography, mass spectrometry

## Abstract

Due to many adverse effects of gestational diabetes mellitus (GDM) on the mother and fetus, its diagnosis is crucial. The presence of GDM can be confirmed by an abnormal fasting plasma glucose level (aFPG) and/or oral glucose tolerance test (OGTT) performed mostly between 24 and 28 gestational week. Both aFPG and abnormal glucose tolerance (aGT) are used to diagnose GDM. In comparison to measurement of FPG, OGTT is time-consuming, usually inconvenient for the patient, and very often needs to be repeated. Therefore, it is necessary to seek tests that will be helpful and convenient to diagnose GDM. For this reason, we investigated the differences in fasting serum metabolites between GDM women with abnGM and normal FPG (aGT-GDM group), with aFPG and normal glucose metabolism (aFPG-GDM group) as well as pregnant women with normal glucose tolerance (NGT) being a control group. Serum metabolites were measured by an untargeted approach using gas chromatography–mass spectrometry (GC–MS). In the discovery phase, fasting serum samples collected from 79 pregnant women (aFPG-GDM, *n* = 24; aGT-GDM, *n* = 26; NGT, *n* = 29) between 24 and 28 weeks of gestation (gwk) were fingerprinted. A set of metabolites (α–hydroxybutyric acid (α–HB), β–hydroxybutyric acid (β–HB), and several fatty acids) significant in aGT-GDM vs NGT but not significant in aFPG-GDM vs NGT comparison in the discovery phase was selected for validation. These metabolites were quantified by a targeted GC–MS method in a validation cohort consisted of 163 pregnant women (aFPG-GDM, *n* = 51; aGT-GDM, *n* = 44; and NGT, *n* = 68). Targeted analyses were also performed on the serum collected from 92 healthy women in the first trimester (8–14 gwk) who were NGT at this time, but in the second trimester (24–28 gwk) they were diagnosed with GDM. It was found that α–HB, β–HB, and several fatty acids were associated with aGT-GDM. A combination of α–HB, β–HB, and myristic acid was found highly specific and sensitive for the diagnosis of GDM manifested by aGT-GDM (AUC = 0.828) or to select women at a risk of aGT-GDM in the first trimester (AUC = 0.791). Our findings provide new potential markers of GDM and may have implications for its early diagnosis.

## Introduction

Gestational diabetes mellitus (GDM), the most common form of metabolic complication in pregnancy (Tenenbaum-Gavish et al., 2020), is defined as any degree of glucose intolerance with the onset or first recognition during pregnancy (Sweeting et al., 2019). GDM affects from 2 to 38% of pregnancies, depending on the diagnostic criteria and population studied (Alesi et al., 2021). Additionally, its prevalence worldwide is rising (Mdoe et al., 2021). In 2017, GDM affected about 204 million women worldwide, with a projection to increase to 308 million by 2045, mostly in developing countries (Yahaya et al., 2020). Several factors can impact the onset of GDM, including immune function disorder, heredity, gene mutations, and especially the effect of hormones (Mdoe et al., 2021). Women who had GDM have an elevated risk to develop diabetes mellitus type 2 (T2DM) or cardiovascular diseases, as well as obesity or hyperlipidemia in later life (Plows et al., 2018). Consequently, the early diagnosis of GDM could be crucial to prevent abovementioned disorders (Buchanan et al., 2012).

Both conditions, abnormal fasting plasma glucose (aFPG) or abnormal results of oral glucose tolerance test (OGTT), which is an indicator of abnormal glucose tolerance (aGT), are used to diagnose GDM. According to Smirnakis et al. (2005) and Riskin-Mashiah et al. (2009), evaluation of fasting plasma glucose (FPG) in the early pregnancy can be used to indicate women at risk for GDM before the 24th week of gestation (gwk). However, recent studies have shown that FPG in early pregnancy was a poor predictor of GDM (Benhalima et al., 2021; Cosson et al., 2021). On the other side, OGTT, in comparison to the single fasting blood collection needed for an FPG measurement, is time-consuming, inconvenient, and may induce nausea and vomiting in some patients (Cosson et al., 2017). However, it is still a “gold standard” for GDM diagnosis (Bogdanet et al., 2020). Finally, even if an abnormal result for FPG is observed in early pregnancy, the OGTT procedure very often needs to be repeated at 24 gwk, which can be refused by some women (Cosson et al., 2017). Consequently, markers allowing for the diagnosis of GDM manifested solely by aGT, without performing OGTT, are needed. Currently an OGTT screening procedure, according to the International Association of Diabetes and Pregnancy Study Groups (IADPSG) criteria (Gupta et al., 2015), should take place between 24 and 28 gwk. Diagnostic or prognostic markers to indicate GDM presence or risk of future development in the early pregnancy are urgently needed. Early diagnosis may allow introduction of effective prevention and care strategies, which may ultimately reduce complications associated with GDM (Brink et al., 2016).

Recent findings have highlighted metabolomics as a prime candidate for evaluating potential markers for GDM (Mao et al., 2017) because of its capacity to detect early deregulations and disruptions in metabolism associated with different diseases (Mojsak et al., 2021). Therefore, it can be used as a potential tool to determine a metabolite or a set of metabolites allowing diagnosis or prediction of GDM (Sakurai et al., 2019). According to reviewed literature reports, several predictive biomarkers of GDM have been suggested, e.g., specific micro-RNAs, amino acids, fatty acids, triglycerides, phosphatidylcholines, or carbohydrates, pyroglutamic, glutamic, phenylacetic and pantothenic acids, xanthine or proteins such as adiponectin, visfatin, omentin-1, fatty acid–binding protein-4, retinol-binding protein-4, globulin, afamin, or fetuin-A (Enquobahrie et al., 2015; Lu et al., 2016; Zhao et al., 2018; Lorenzo-Almorós et al., 2019; Tenenbaum-Gavish et al., 2020; Tian et al., 2021). Numerous serum or plasma metabolites such as α–hydroxybutyric acid (α–HB) (Dudzik et al., 2017), β–hydroxybutyric acid (β–HB) (Scholtens et al., 2014; Dudzik et al., 2017), amino acids (Scholtens et al., 2014; Enquobahrie et al., 2015), sugars (Enquobahrie et al., 2015), and fatty acids (Enquobahrie et al., 2015; Dudzik et al., 2017) have shown to be associated with this disease using various approaches such as gas chromatography–mass spectrometry (GC–MS) (Dudzik et al., 2017; Scholtens et al., 2014; Rahman et al., 2018; O’Neill et al., 2018), liquid chromatography–mass spectrometry (LC–MS) (Liu et al., 2016; Hou et al., 2018; Tian et al., 2021), and nuclear magnetic resonance (NMR) spectroscopy (Pinto et al., 2015; Hou et al., 2018). GC-MS is adequately sensitive to detect subtle differences in the level of serum/plasma metabolites (Dudzik et al., 2017) and was used in the present study.

However, until now, metabolomics studies on GDM were focused on case-control studies, in which the case group comprised women diagnosed with GDM (Pinto et al., 2015; Liu et al., 2016; Hou et al., 2018). There is a lack of studies in which GDM women were divided into separate subgroups depending on the diagnostic scenario, i.e., women with aGT and normal FPG (aGT-GDM group) and women with aFPG and normal glucose tolerance (aFPG-GDM group). To the best of our knowledge, this is the first study conducted to seek differences in metabolic profiles between the abovementioned GDM subgroups of patients and a control group with normal FPG and glucose metabolism. Such an approach has the potential to find the relevance of metabolomics in diagnosis of GDM.

## Materials and Methods

### Study Group

Pregnant women (662) were screened for GDM at the Department of Endocrinology, Diabetology, and Internal Medicine (Medical University of Bialystok, Poland) between 2015 and 2017. For all participants between 24 and 28 gwk, OGTT (75 g) was performed after an overnight fast, with blood samples collected at fasting, 1, and 2 h time points. After clotting at room temperature, fasting serum samples were centrifuged and then separated and frozen at –80°C until the metabolomics assays.

Women were diagnosed with GDM if one of the following criteria was met: fasting glucose ≥92 mg/dl, 1 h glucose ≥180 mg/dl, or 2 h glucose ≥153 mg/dl (Metzger et al., 2010). Women were classified as the aGT-GDM group if they met the following criteria: fasting glucose <92 mg/dl, 1 h glucose ≥180 mg/dl, and/or 2 h glucose ≥153 mg/dl, whilst women were classified as the aFPG-GDM group if they met the following criteria: fasting glucose ≥92 mg/dl, 1 h glucose <180 mg/dl, and 2 h glucose <153 mg/dl. The control group (NGT) comprised participants with the following criteria: fasting glucose <92 mg/dl, 1 h glucose <180 mg/dl, and 2 h glucose <153 mg/dl. All women were characterized by a normal (<5.7%) (Bozkurt et al., 2020) glycated hemoglobin (HbA1c) level.

**FIGURE 1:**
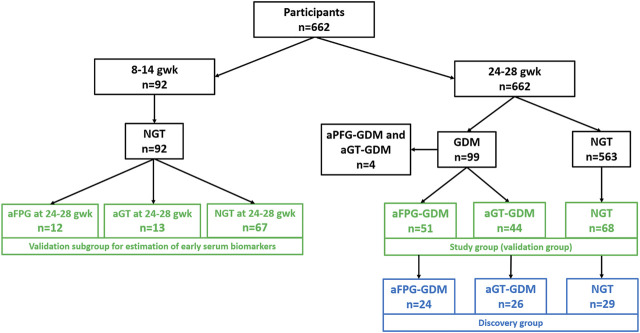
Flow chart presenting participants’ selection. Samples selected for the discovery phase are presented in blue rectangles, while those for the validation stage, in green rectangles.

From the total number of 662 participants, 99 women were diagnosed with GDM between 24–28 gwk; among them, 44 individuals were classified as aGT-GDM, 51 as aFPG-GDM, and only four (excluded from this study) met the criteria to be classified to both–GDM groups. Women from an aGT-GDM group (*n* = 44) and aFPG-GDM group (*n* = 51) together with 68 women selected from the NGT group formed a study group (*n* = 163) which was also a validation cohort. From each subgroup of the validation cohort age- and BMI-matched women were selected for the discovery cohort. A discovery cohort comprised 24 women with aFPG-GDM, 26 with aGT-GDM, and 29 with NGT. Moreover, for the limited set of women (*n* = 92) fasting serum samples in the first trimester (8–14 gwk) were collected. At that period, all of the selected subjects were characterized by the normal fasting glucose level. However, between 24–28 gwk, some of these women were diagnosed with aGT-GDM (*n* = 13), others with aFPG–GDM (*n* = 12), and the rest remained NGT (*n* = 67). These subjects (*n* = 92) were included in the present study as the additional independent validation cohort (Supplementary Table S1). A flow chart showing classification of participants into specific study groups is presented on Figure 1, while the detailed anthropometric and metabolic characteristics of the groups are listed in Table 1.

**TABLE 1:** Anthropometric and metabolic characteristics of the subgroups–discovery and validation cohort–second trimester (24–28 weeks of gestation).

Participants’ characteristics	Discovery cohort			Validation cohort		
	NGT	aGT-GDM	aFPG-GDM	NGT	aGT-GDM	aFPG-GDM
N	29	26	24	68	44	51
Age [years]	29 (5)	33 (6)*^,a^	28 (7)	28 (4)	32 (6)^**,a^	29 (7)
Maternal prepregnancy BMI [kg/m^2^]	23.4 (3.7)	23.9 (5.7)	22.4 (7.7)	22.2 (3.2)	22.8 (6.7)	23.2 (5.3)
Maternal pregnancy BMI [kg/m^2^]	27 (5)	26.4 (5.4)	26.2 (6.9)	25.6 (3.2)	25.65 (6.4)	26.2 (5)
BMI gain	2.7 (1.6)	2.8 (2.3)	3.4 (1.6)	2.9 (1.8)	2.6 (2.2)	2.8 (2.2)
Total cholesterol [mg/dL]	232 (81)	245.5 (72)	238.5 (54)	231 (48.5)	243 (57)	236 (62)
LDL cholesterol [mg/dL]	130.4 (71)	123 (70.1)	127.7 (48)	111.8 (53.6)	132 (39.6)^*^	129 (66.2)^a^
Triglycerides [mg/dL]	183 (68)	173 (60.8)	197.5 (105)	146.5 (55)	185 (89)*	163.5 (79.3)
HDL cholesterol [mg/dL]	73 (21.4)	72.5 (29)	68.5 (23)	90 (28)	77.5 (26.5)^a^	72.5 (23.8)^**^
HbA1c (%)	4.7 [0.4]	4.8 [0.4]	4.9 [0.5]	4.7 [0.3]	4.9 [0.3]*	4.9 [0.4]*
HbA1c (mmol/mol)	28 [4.4]	29 [4.4]	30 [5.5]	28 [3.3]	30 [3.3]*	30 [4.4]*
Fasting plasma glucose [mg/dL]	75 (8)	84 (7)*^,b^	94 (4.2)^**^	81 (5.5)	84 (6.3)^*,b^	94 (4)**
Glucose 1 h [mg/dL]	133 (39)	187 (16.3)**^,b^	138 (24)	114.5 (41.3)	184 (18.8)**^,b^	131 (26.5)*
Glucose 2 h [mg/dL]	109.5 [19.6]	154 [21.5]**^,b^	116 [22.8]	104.5 [19.4]	156.6 [22]**^,b^	111.5 [20.6]
Fasting insulin [μIU/mL]	10.5 (6.4)	14.3 (7.1)*	16.4 (13)*	10.9 (4.3)	13.2 (7.8)*	15.5 (10.7)**
HOMA–IR	2 (1.1)	2.9 (1.6)*^,a^	3.7 (3.1)**	2.2 (0.9)	2.73 (1.9)*	3.5 (2.7)**
HOMA%β	297.7 (201.5)	279.9 (112.1)^a^	193.6 (144.5)*	211.4 (103.6)	253.6 (109)^a^	169 (116.5)*
QUICKI	0.3 (0.03)	0.3 (0.02)*^,a^	0.3 (0.03)**	0.3 (0.02)	0.3 (0.03)*^,a^	0.3 (0.03)**

Data are presented as mean [SD] or median (interquartile range). Abbreviations: NGT, normal glucose tolerance; aGT–GDM, group with diagnosed GDM, based on abnormal OGTT, aFPG–GDM–group with abnormal fasting plasma glucose, HOMA, homeostatic model assessment; IR, insulin resistance; QUICKI, quantitative insulin–sensitivity check index. Statistical significance for NGT *vs* aGT-GDM, and NGT *vs* aFPG-GDM, comparisons: * *ƿ* < 0.05, ** *ƿ* < 0.0001. Statistical significance for aGT-GDM *vs* aFPG-GDM, comparison: ^a^ *ƿ* < 0.05,^b^ *ƿ* < 0.0001. Continuous data of clinical characteristics were analyzed by Student’s t–test for normally distributed data or by the Mann–Whitney *U* test for the data without the normal distribution.

### GC–MS-Based Metabolomics

Untargeted and targeted metabolomics analyses were performed on the GC system (Agilent Technologies 7890B) consisting of an autosampler (MultiPurpose Sampler, Gerstel, Germany) and an accurate-mass Q-TOF (Agilent Technologies 7200) detector. Derivative samples (1 μL) were injected into a GC column DB5–MS (30 m length, 0.250 mm i.d., 0.25 μm film 95% dimethyl/5% diphenylpolysiloxane) with a pre–column (10 m J&W integrated with Agilent 122–5532G). The temperature gradient was programmed at 60 °C (held for 1 min), with a ramping increase rate of 10 °C/min up to 325°C (held for 10 min). The total analysis time was 37.5 min. The EI source was operated at 70 eV. The method was RT locked at 19.663 min (elution time of the internal standard–methyl stearate). The mass spectrometer was operated in the scan mode over a mass range of *m*/*z* 45–600 at a rate of 10.00 spectra/s. A detailed description of used reagents and applied analytical conditions is available in the Supplementary Materials File.

Extraction of serum metabolites was performed as described previously (Mojsak et al., 2021). The derivatization procedure was carried out in two steps. For methoximation, 10 μL of O–methoxyamine hydrochloride (15 mg/ml) in pyridine was added to each vial and vortexed vigorously. The vials were incubated in darkness at room temperature for 16 h. Then, 10 μL of BSTFA with 1% TMCS (v/v) was added, and samples were vortexed for 5 min; silylation was carried out for 1 h at 70°C, and finally, 100 μL of C18:0 methyl ester (10 mg/L in heptane) was added as an internal standard. Samples were mixed again by vortexing gently.

The description of untargeted and targeted GC–MS data treatment is available in the Supplementary Materials File.

## Statistical Analysis

Multivariate methods such as principal component analysis (PCA) and partial least squares–discriminant analysis (PLS–DA) were used for data visualization. PCA and PLS–DA models were built using SIMCA–P+ software (13.0.3.0 Umetrics). Statistical significance of the PLS–DA model was validated with permutation testing.

Distribution of the data was assessed by the Shapiro–Wilk test. Student’s t–test was used for normally distributed data, whilst the Mann–Whitney *U* test was used for nonparametric data. Benjamini–Hochberg *post hoc* corrections were performed. The threshold for statistical significance was 0.05. Statistical analysis was performed by in-house built scripts for MATLAB (7.10.0.499, MathWorks, Natick, MA, United States). Considering the criteria of the Metabolomics Standards Initiative (Fiehn et al., 2007; Salek et al., 2013), all statistically significant metabolites were identified with the highest confidence level (grade 1). Discovery cohort and both validation cohorts were analyzed independently.

Receiver operating characteristic (ROC) analysis was performed using MedCalc ver. 18 (MedCalc Software, Ostend, Belgium). The performance of the models was compared by applying the nonparametric method of Delong et al. (1988). The specificity and sensitivity were determined according to the sample class prediction using the 7-fold cross-validation predicted values of the fitted *Y–predcv* (implemented in SIMCA–P+ software) for observations in the model.

## Results

First, we used GC–MS in an untargeted approach (metabolic fingerprinting) to investigate the differences between aGT-GDM, aFPG-GDM, and NGT groups in the second trimester. Metabolic fingerprinting resulted in a total number of 96 compounds. After data filtering, the matrix was reduced to 50 compounds. As it can be seen in Supplementary Figure S1, quality control samples are tightly clustered on the PCA model (panel A), whereas between-group discrimination is displayed (panel B) on the validated (panels C and D) PLS-DA model. In order to evaluate statistically significant differences between the groups aGT-GDM vs NGT, aFPG-GDM vs NGT, and aGT-GDM vs aFPG-GDM, the univariate statistics was performed. The list of 31 statistically significant metabolites is displayed in Supplementary Table S2. Metabolites significantly discriminating study groups mainly belong to fatty acids, hydroxy acids, and organooxygen compounds. Only four metabolites (mannitol, cetyl alcohol, arabitol, and p-cresol) were found significantly different in the aFPG-GDM vs NGT comparison. Considering the comparison of aGT-GDM and NGT groups, a great number of compounds was represented by increased saturated fatty acids (caprylic 1.46–fold, capric 2.5–fold, lauric 2.04–fold, myristic 1.81–fold, palmitic 1.46–fold, stearic 1.62–fold, heptadecanoic 1.82–fold, and nonanoic 1.68–fold) and increased unsaturated fatty acids (palmitoleic 1.6–fold, oleic 1.73–fold, and linoleic 1.81–fold) in the aGT-GDM group. Another noticeable group of compounds increased in the subjects with aGT-GDM compared to NGT consisted of hydroxy acids and derivatives, with α–HB and β–HB as the most represented (1.28–fold and 1.76–fold change, respectively).

Fourteen of the most promising metabolites, according to the experimental data and literature (Scholtens et al., 2014; Cobb et al., 2015; Dudzik et al., 2017), significantly discriminating an aGT-GDM group from the NGT group, were chosen for quantification in both validation cohorts. Metabolites found as significant in the validation study for NGT vs aGT-GDM comparison in any of validation cohorts are presented in Table 2.

**TABLE 2:** Statistically significant metabolites for NGT vs aGT-GDM comparison based on the validation study results.

Metabolite	1st trimester			2nd trimester		
	NGT	aGT-GDM	aFPG-GDM	NGT	aGT-GDM	aFPG-GDM
α-Hydroxybutyric acid [mg/L]	1.45 (0.35)	1.8 (0.45)*	1.54 (0.3)	1.26 (0.25)	1.42 (0.24)***	1.36 (0.32)
Β-Hydroxybutyric acid [mg/L]	1.28 (0.72)	1.63 (0.71)*	1.45 (0.44)	1.29 (0.46)	1.69 (1.07)**	1.47 (0.68)
Capric acid [mg/L]	0.24 (0.07)	0.32 (0.08)*	0.26 (0.07)	-	-	-
Nonanoic acid [mg/L]	-	-	-	0.24 (0.08)	0.27 (0.11)**	0.26 (0.11)
Lauric acid [mg/L]	0.21 (0.09)	0.28 (0.11)*	0.26 (0.08)	0.23 (0.08)	0.28 (0.11)**	0.24 (0.15)
Myristic acid [mg/L]	0.42 (0.27)	0.59 (0.13)*	0.57 (0.21)	0.47 (0.23)	0.69 (0.25)****	0.53 (0.3)
Palmitic acid [mg/L]	13.32 (2.85)	14.75 (1.97)*	13.52 (2.67)	13.41 (2.34)	14.93 (2.7)***	14.18 (2.36)
Oleic acid [mg/L]	38.77 (23.19)	47.7 (33.45)*	45.6 (23.73)	40.33 (16.36)	48.45 (18.15)**	39.55 (18.63)

Classification of the subgroups in the 1^st^ trimester study group was based on the OGTT results obtained in the 2^nd^ trimester. Data are presented as a median and interquartile range in brackets. Statistical significance for aGT–group vs NGT, comparison: * - *ƿ* <0.05, ** - *ƿ* <0.01, *** - *ƿ* <0.0001, **** - *ƿ* <0.00001 by Mann–Whitney *U* test. Abbreviations: NGT, normal glucose tolerance; aGT-GDM, group with diagnosed GDM, based on abnormal OGTT, aFPG-GDM, group with abnormal fasting plasma glucose.

**FIGURE 2:**
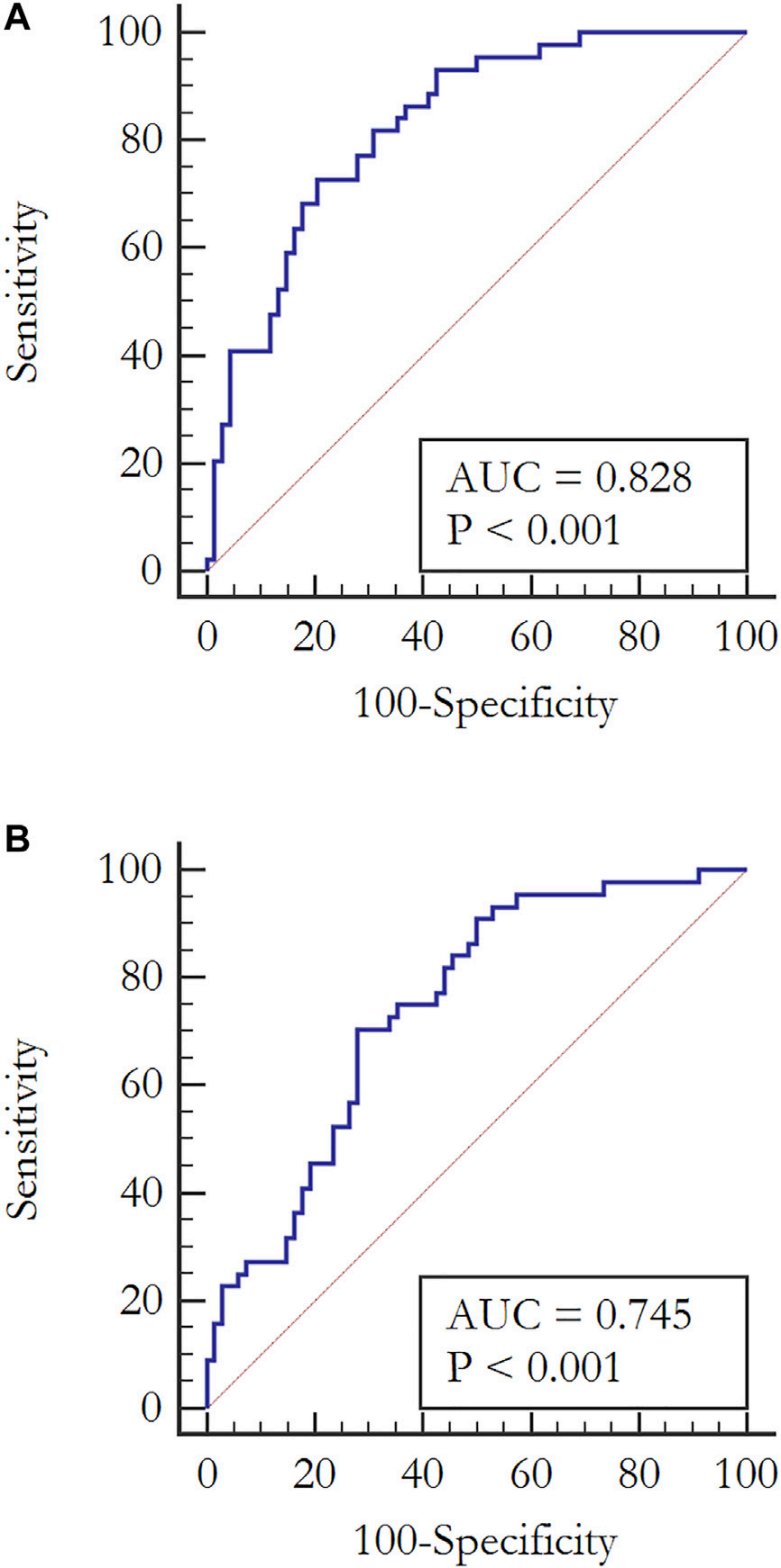
ROC curves analyses evaluating the clinical usefulness of potential biomarkers to diagnose pregnant women with aGT–group from a fasting serum sample in the second trimester. **(A)** Combination of α–HB, β–HB, and myristic acid: AUC = 0.828, CI (0.745–0.892), Sensitivity = 72.7, Specificity = 79.4, *p* < 0.0001 **(B)** α–HB: AUC = 0.745, CI (0.654–0.823), Sensitivity = 70.5, Specificity = 72, *p* < 0.0001.

We observed an increased level for all of the metabolites in the subjects with the aGT-GDM group in comparison to NGT individuals, which also confirms the results of fingerprinting analysis. Interestingly, the majority of compounds (i.e., α–HB, β–HB, myristic, lauric, palmitic, and oleic acids) were statistically significant and shared a similar change in the concentration level between the aGT–group and NGT in both the first and second trimester. The only difference between the trimesters was found for nonanoic acid and capric acid, statistically significant only in the second or first trimester, respectively. α–HB (*p* = 0.00005) and myristic acid (*p* = 0.000005) were found to be strongly associated with the aGT–group. To evaluate the clinical usefulness and predictive ability of potential biomarkers to distinguish the aGT–group from NGT, a ROC curve analysis was performed for all of the metabolites that passed the validation independently as well as for the combinations of different metabolites (Supplementary Table S3). Considering each metabolite independently, the best predictive power to discriminate aGT-GDM patients, characterized by fair accuracy of the test, was found for myristic acid (Area Under Curve, AUC = 0.787 in the second trimester and AUC = 0.759 in the first trimester), α–HB (AUC = 0.745 in the second trimester and AUC = 0.797 in the first trimester), and palmitic acid (AUC = 0.754 in the second trimester and AUC = 0.745 in the first trimester). The ROC curve and the corresponding AUC were significantly improved when combining the selected metabolites into different models. The combination of fatty acids myristic, lauric, palmitic, oleic, and nonanoic (in case of the second trimester) or capric (first trimester) acid was found to have a good predictive ability (AUC = 0.775 in the second trimester and AUC = 0.747 in the first trimester). Furthermore, an addition of α–HB and β–HB to the combination of fatty acids improved its predictive value (AUC = 0.815 in the second trimester and AUC = 0.772 in the first trimester) (Supplementary Table S3). However, the best diagnostic power considering its accuracy, sensitivity, and specificity was found for the model consisting of α–HB, β–HB, and myristic acid (AUC = 0.828 in the second trimester and AUC = 0.791 in the first trimester) (Figure 2).

## Discussion

The discussion on the most appropriate screening strategy for GDM with OGTT at 24–28 weeks of pregnancy is ongoing (Gupta et al., 2015). Detection in the early pregnancy of metabolites showing subtle metabolic perturbations indicating GDM presence or risk of development has clinical significance for early diagnosis or prognosis (Tenenbaum-Gavish et al., 2020), which is crucial to prevent subsequent damage in both the mother and fetus (Brink et al., 2016). Metabolomics research can not only propose novel diagnostic or prognostic GDM biomarkers but may also allow monitoring of pregnancy complications for better GDM management (Donovan et al., 2018).

Therefore, in the discovery phase of this study, we have evaluated differences in serum metabolic profiles between the patients with GDM diagnosed solely with aFPG or aGT in comparison to pregnant women with NGT. Among significant metabolites (Table 2), mainly fatty acids (palmitic, stearic, capric, lauric, oleic, caprylic, myristic, nonanoic, heptadecanoic, and palmitoleic acids) and both hydroxybutyric acids (α and β) were observed. The same metabolites or metabolites from the same classes have already been proposed by other authors as characteristic to GDM. For instance, in the study conducted by (Hou et al., 2018), almost a half of FFAs were elevated in GDM patients. Dudzik et al. (2017) reported an increased level of several fatty acids in the GDM group compared to NGT, with stearic acid as the most represented. Enquobahrie et al. (2015) presented the results of untargeted GC–MS analysis of serum samples collected in the early pregnancy. Out of 17 discovered metabolites distinguishing GDM from NGT individuals, myristic and oleic acids were among the most abundant metabolites within the GDM group. Despite the fact that the diagnostic criteria used by Enquobahrie et al. (2015) were different than in the presented study, the results for myristic acid are consistent with ours.

Oxidation of free fatty acids and excess acetyl-CoA production lead to an increase in the β–HB level (Lu et al., 2021). Increased levels of α–HB and β–HB in GDM patients in comparison to those of NGT women were also observed by others. In the recent study conducted by Lu et al. (2021) on the Chinese population, an elevated level of β–HBA in the second or third trimester was found associated with GDM. In the already mentioned study of Dudzik et al. (2017), increased levels of α–HB and β–HB in the GDM group as compared to NGT were also noted. Moreover, Scholtens et al. (2014) demonstrated broad-scale perturbations in hyperglycemic pregnant women and compared metabolic profiles of mothers with high and low FPG levels. Among significant metabolites, α–HB and β–HB were noted. The study was focused largely on the differences between high and normal fasting plasma glucose subjects. Nevertheless, according to the clinical characteristics presented in this report, among the individuals defined as high–FPG, subjects with increased plasma glucose level at 1 h or 2 h in OGTT were also present.

The elevated level of α–HB can be associated with oxidative stress or increased insulin resistance (Meigs et al., 2007). Oxidative stress is a result of enhanced mitochondrial activity. To manage the resulting oxidative stress, glutathione biosynthesis is activated, and consequently, a demand for cysteine is increased. During the conversion of cystathionine to cysteine, α–ketobutyric acid (α–KB) is produced, whereas α–HB is a by–product of α–KB formation (Dudzik et al., 2014). Another important metabolite associated with aGT–GDM individuals is β–HB. Besides its known role as an important ketone body, which carries energy from the liver to peripheral tissues during fasting or exercise, β–HB plays a significant role in cellular processes regulation by altering the level of other regulatory metabolites such as acetyl-CoA, succinyl-CoA, and NAD^+^ (Newman and Verdin, 2014). Moreover, insulin resistance is characterized by increased lipolysis and increased fatty acid oxidation (Bronisz et al., 2018). IR is observed in normal pregnancy, but in the case of excessive IR and significant β-cell dysfunction, GDM develops (Chen et al., 2019; Kampmann et al., 2019). Increased circulating free fatty acids (also observed in our study) have been recognized as one of the most critical factors contributing to IR and altering insulin secretion (Chen et al., 2019).

However, none of the abovementioned metabolomics studies on GDM considered the differences among the women diagnosed solely with either aGT or aFPG. These two distinct metabolic states, but described as isolated impaired glucose tolerance (iIGT) and isolated impaired fasting glucose (iIFG), were previously investigated in pre-T2DM nonpregnant individuals (Gall et al., 2010; Ferrannini et al., 2013; Cobb et al., 2015; Cobb et al., 2016). These reports demonstrate some consistency with the results of our study, particularly for iIGT individuals. For instance, Gall et al. (2010) proposed that α–HB can serve as an early biomarker of insulin resistance and IGT in nondiabetic individuals. Its increased level was associated with increased lipid oxidation and oxidative stress. Furthermore, the role of α–HB in the pathophysiology of the prediabetes state was proved by Cobb et al. (2015; 2016). Besides the elevated concentration of α–HB in the individuals with IGT, they also found an increase of β–HB together with an increased free fatty acids level, which supports the concept of using α–HB, β–HB, and free fatty acids as biomarkers of iIGT without performing an OGTT. As the aim of this study was to find biomarkers that could replace OGTT, but in the case of GDM diagnosis, we evaluated the diagnostic potential of metabolites statistically significant for the aGT-GDM vs NGT comparison using data obtained in the validation phase. It was confirmed that a combination of α–HB, β–HB, and myristic acid was highly specific and sensitive for the diagnosis of GDM manifested by abnormal glucose tolerance with AUC = 0.828 (Figure 2).

Samples belonging to the other validation group were collected in the first trimester (8–14 gwk) from women with normal FPG. However, some of these women (Figure 1) were diagnosed with GDM between 24–28 gwk. Performed targeted analyses revealed a similar metabolite profile in the first and the second trimester of pregnancy, considering the change in the concentration level of significant metabolites between aGT-GDM and NGT individuals. Despite the fact that the number of samples from the first trimester was limited, the comparable tendency in both time points of pregnancy shows that α–HB, β–HB, and myristic acid may serve as early biomarkers of later-onset GDM (AUC = 0.791, Table S3). However, we are aware that normoglycemic women in the first trimester did not undergo OGTT. According to the diagnostic strategy (International Association of Diabetes and Pregnancy Study Groups Consensus Panel et al., 2010), if the fasting plasma glucose level at the first prenatal visit is below 92 mg/dl, women should be screened for GDM with 75 g OGTT between 24 and 28 gwk. Therefore, because of a lack of data, we cannot reject the possibility of the already existing aGT-GDM. Further investigations are needed to evaluate whether the proposed markers are strictly related to the presence of IFG or can be considered predictive. Nevertheless, diagnosing individuals at high risk would potentially allow the prevention of GDM development by implementing lifestyle modifications with adequate diet and physical activity (Tobias et al., 2011; Zhang et al., 2016).

Based on the literature review, there are only few reports in the literature (Ravnsborg et al., 2016; Leitner et al., 2017; Corcoran et al., 2018; Yin et al., 2018) where the GDM predictive metabolites found in metabolomics are subjected to further validation. For example, Leitner et al. (2017) received similar results for α–HBA and β–HBA as strong markers in the prediction of GDM. This hypothesis was additionally tested by targeted profiling of serotonin-derived metabolites, also in urine samples, and went one step further with the integration of plasma and urine metabolic markers to improve the prediction accuracy of GDM in this study. Due to this fact, the continuation of our study should be the replication of the findings in a large cohort study and developing methods for other matrices, which may improve the understanding of GDM pathogenesis and may have implications for its early diagnosis.

## Conclusion

Our study explored differences in the serum metabolic profile in pregnancy, firstly by untargeted, and finally by quantitative analysis with the GC–MS technique. In the first part of the study, we identified and confirmed a set of metabolites representative for GDM women with abnormal glucose tolerance but a normal FPG level (aGT-GDM group). A combination of three metabolites (α–HB, β–HB, and myristic acid) was found strongly associated with aGT-GDM. Measurement of the concentrations of the proposed panel of metabolites in the fasting serum sample has the potential to be a useful clinical test to diagnose GDM in the second trimester of pregnancy without the need to perform OGTT. Moreover, these metabolites can potentially be used to identify, in the early pregnancy, subjects with aGT-GDM or at high risk for developing GDM manifested by abnormal glucose metabolism in the near future. The proposed panel of metabolites can potentially be used instead of OGTT. However, measurement of FPG is still needed to indicate women with aFPG-GDM. Consequently, fasting plasma glucose measurement should be accompanied by the measurement of α–HB, β–HB, and myristic acid in the fasting serum sample. From the perspective of pregnant women, it will facilitate the diagnostic procedure, as only a single fasting blood collection will be needed. Measurement of these GDM markers can be easily performed using a method based on chromatographic separation and MS detection. The application of MS in clinical laboratories has developed very well in the last decade, and this technology is already used for such routine applications as therapeutic drug monitoring, newborn screening, or steroid analysis (Honour et al., 2018; Cui et al., 2020; Seger and Salzmann, 2020). Consequently, MS combined with a separation technique can be easily adapted to measure metabolites significant in this study. Our work contributes to the design of novel diagnostic targets that may facilitate precision medicine and lead to the development of personalized diagnostics of aGT-GDM based on the three biomarkers (α–HB, β–HB, and myristic acid).

## Data Availability

The original contributions presented in the study are included in the article/Supplementary Files; further inquiries can be directed to the corresponding author.
