# Supplementary material for: Gas Chromatography–Mass Spectroscopy-Based Metabolomics Analysis Reveals Potential Biochemical Markers for Diagnosis of Gestational Diabetes Mellitus
Source: Front Pharmacol. 2021 Nov 19;12:770240. doi: 10.3389/fphar.2021.770240 (PMC8640240; doi:10.3389/fphar.2021.770240)
Supplement: Supplementary file 1 [file DataSheet1.PDF]

Supplementary materials to:

Gas Chromatography–Mass Spectroscopy based metabolomics analysis reveals potential biochemical markers for diagnosis of gestational diabetes mellitus

Beata A. Raczkowska[1], Patrycja Mojsak[1], David Rojo[2], Beata Telejko[§3], Magdalena Paczkowska–Abdulsalam[1], Justyna Hryniewicka[3], Anna Zielinska–Maciulewska[3], Malgorzata Szelachowska[3], Maria Gorska[3], Coral Barbas[2], Adam Kretowski[1,3], Michal Ciborowski[1]*

[1]Clinical Research Centre, Medical University of Bialystok, Bialystok, 15–276, Poland
[2] Centro de Metabolómica y Bioanálisis (CEMBIO), Facultad de Farmacia, Universidad CEU San Pablo, Campus Montepríncipe, Madrid, 28660, Spain
[3]Department of Endocrinology, Diabetology and Internal Medicine, Medical University of Bialystok, Bialystok, 15–276, Poland
[§]Deceased 28 October 2017.

***corresponding author: michal.ciborowski@umb.edu.pl**

Metabolomics measurements

1. Reagents.

The following reagents and standards were used: MilliQ® water (Millipore, Billerica, MA, USA), heptane (Sigma–Aldrich, Steinheim, Germany), methanol (MeOH) (Sigma–Aldrich, Steinheim, Germany), ethanol (EtOH) (Sigma–Aldrich, Steinheim, Germany), pyridine (Sigma–Aldrich, Steinheim, Germany), O–methoxyamine hydrochloride (Sigma–Aldrich, Steinheim, Germany) and N,O–bis(trimethylsilyl) trifluoroacetamide (BSTFA) plus 1% trimethylchlorosilane (TMCS) (Pierce Chemical Co, Rockford, IL, USA). Stearic acid methyl ester (C18:0 methyl ester) (Sigma–Aldrich, Steinheim, Germany) was used as internal standard for GC–MS. A FAME mix (fatty acid methyl esters, e.g. caprylic acid, capric acid, lauric acid, tridecanoic acid, myristic acid, oleic acid, pentadecanoic acid, palmitic acid, palmitoleic acid, heptadecanoid acid, stearic acid, elaidic acid, oleic acid, linoleic acid, arachidic acid, cis–11–eicosenoic acid, linolenic acid, behenic acid and erucic acid) was purchased from Supelco

(Bellefonte, PA, USA). The standards for quantification (α–hydroxybutyric acid, β–hydroxybutyric acid, benzoic acid, caprylic acid, nonanoic acid, capric acid, lauric acid, myristic acid, palmitoleic acid, palmitic acid, heptadecanoic acid, linoleic acid, oleic acid and stearic acid) were purchased from Sigma–Aldrich (Steinheim, Germany) and the deuterated compounds (4–hydroxybutyric acid D6, benzoic acid D5, lauric acid D23, oleic acid D2) from Cambridge Isotope Laboratories (Andover, MA, USA).

2. GC settings

2.1 Untargeted analysis.

The helium carrier gas flow rate was set at 1.56 mL/min and the injector temperature at 250°C. The split ratio was fixed at 1:12 with 18.71 mL/min helium split flow into an Agilent 5190–2295 Ultra Inert Inlet Liner. The analytical run was set up starting with the analysis of seven samples to equilibrate the chromatographic system; calibration of the TOF–MS equipment was performed between blocks of three injections.

2.2 Target analysis of metabolites.

The helium carrier gas flow rate was set at 0.78 mL/min and the injector temperature at 250°C. The split ratio was fixed at 1:25 with 19.5 mL/min helium split flow into an Agilent 5190–2295 Ultra Inert Inlet Liner. The analytical run was set up as follows: firstly, the analysis of seven injections to equilibrate the chromatographic system, followed by the analytical samples in a randomized order. The series of calibration samples (containing the isotopically labelled standards) were injected at the beginning of the sequence and after the final analytical sample was injected. The TOF–MS equipment was calibrated between blocks of five injections.

3. GC–MS data treatment.

3.1 Untargeted data analysis.

The deconvolution and identification was performed using Mass Hunter Quantitative Unknowns Analysis software (B.07.00, Agilent), alignment with Mass Profiler Professional software (version 13.0, Agilent) and peak integration using Mass Hunter Quantitative Analysis software (version B.07.00, Agilent). The identification was performed based on FiehnLib (33) and NIST 14 libraries. In order to perform the differential analysis on the metabolomics data, the variables were then filtered as proposed by Godzien et al. (34), with minor modifications. Missing values were replaced by k–means nearest neighbour (35) using in–house built scripts for MATLAB (7.10.0.499, MathWorks, Natick, MA, USA).

3.2 Analysis of targeted data and quantification of metabolites.

Serum samples for targeted analysis were prepared analogically to fingerprinting analysis. Additionally, series of calibration samples were prepared from plasma extracts spiked with different quantities of 4–hydroxybutyric acid D^6, benzoic acid D^5, lauric acid D^{23}, oleic acid D^2. Targeted analysis was performed on the GC system (Agilent Technologies 7890B) consisted of an autosampler (MultiPurpose Sampler, Gerstel, Germany) and an accurate–mass Q–TOF (Agilent Technologies 7250) detector. Derivatised samples (1 µL) were injected into the same GC–Column as in the discovery phase.

The corresponding peak areas were integrated by MassHunter Quantitative Analysis (B.08.00, Agilent). The final concentrations were calculated based on the calibration curves obtained for respective analytical standards. The linearity of the relative response versus concentration was assessed under the same analytical conditions for 4–hydroxybutyric acid D^6 (r=0.997), benzoic acid D^5 (r=0.999), lauric acid D^{23} (r=0.997), and oleic acid D^2 (r=0.997). Finally, the concentration of each analyte was calculated based on the response factor between it and the corresponding deuterated standard: i) 4–hydroxybutyric acid D6 for α–HB and β–

HB; ii) lauric acid D23 for caprylic, nonanoic, capric, lauric and myristic acids; iii) oleic acid D2 for palmitoleic, palmitic, heptadecanoic, linoleic, oleic and stearic acids.

Table S1. Anthropometric and metabolic characteristics of the subgroups- validation cohort – first trimester (8-14 week of gestation).

	NGT	aGT–GDM	aFPG–GDM
n	67	13	12
Age [years]	28 (4)	28 (7)	31 (9)
Maternal pre-pregnancy BMI [kg/m^2]	22.2 (3.2)	21.6 (2.1)	23.5 (2.8)
Maternal pregnancy BMI [kg/m^2]	22.8 (2.6)	22.3 (2.5)	24.8 (2.5)
BMI gain	0.5 [0.7]	0.5 [0.7]	0.7 [0.6]
Total cholesterol [mg/dL]	167.1 [28.4]	177.9 [30.1]	171.1 [41.8]
LDL [mg/dL]	71.1 [21.4]	80.5 [22.9]	87.4 [38.5]
Triglycerides [mg/dL]	87 (42.5)	112 (42)	73.5 (46.8)
HDL [mg/dL]	79.8 [17.9]	78.3 [15]	67.3 [12.5]*
HbA1c (%)	5 [0.3]	5.1 [0.3]	5 [0.5]
HbA1c (mmol/mol)	31 [3.3]	32 [3.3]	31 [5.1]
Fasting plasma glucose [mg/dL]	86 (6)	83 (5)‡	89 (4.3)†
Fasting insulin [μIU/mL]	9.2 (2.8)	10.3 (4.6)	9.2 (3.9)
HOMA-IR	2 (0.7)	2.1 (1.1)	2 (0.9)
HOMA%β	156.9 (64.1)	162.3 (39.4)	132.7 (53.8)†
QUICKI	0.3 (0.02)	0.3 (0.03)	0.3 (0.02)

Data are presented as mean [SD] or median (interquartile range). Abbreviations: NGT - Normal Glucose Tolerance, aGT–GDM – group with diagnosed GDM based on abnormal OGTT, aFPG–GDM– group with abnormal fast plasma glucose, HOMA - homeostatic model assessment, IR-insulin resistance, QUICKI - quantitative insulin-sensitivity check index. Statistical significance; vs NGT: *P < 0.01 by Student's t-test. †P < 0.05 by Mann-Whitney U test. aGT–GDM vs aFPG–GDM: ‡P < 0.01 by Student's t-test. Measurements taken at the 1st trimester (8-14 weeks of gestation).

Supplementary Table S2. Metabolites identified in the fingerprinting study showing between-group differences.

Metabolite	Metabolite subclass (HMDB)	aGT-GDM vs NGT	aFPG–GDM vs NGT	aGT-GDM vs aFPG–GDM
α-Hydroxybutyric acid	Alpha hydroxy acids and derivatives	1.28‖	1.23	1.05
Benzoic acid	Benzoic acids and derivatives	2.62§	0.99	2.65§
β-Hydroxybutyric acid	Beta hydroxy acids and derivatives	1.76†	1.28	1.38
Mannitol	Carbohydrates and carbohydrate conjugates	1.02	1.39‖	0.73‖
Xylitol	Carbohydrates and carbohydrate conjugates	0.92	1.18	0.78†
Aminomalonic acid	Carboxylic acids and derivatives	1.48	1.01	1.46‖
N-acetyl-L-glutamic acid	Carboxylic acids and derivatives	0.67	1.57	0.42‖
Fumaric acid	Dicarboxylic acids and derivatives	1.41‖	1.25	1.13
Palmitic acid	Fatty acids and conjugates	1.46‡	1.07	1.37*
Stearic acid	Fatty acids and conjugates	1.62†	0.98	1.65†
Capric acid	Fatty acids and conjugates	2.5§	1.15	2.17**
Lauric acid	Fatty acids and conjugates	2.04§	1.73	1.18#
Oleic acid	Fatty acids and conjugates	1.73‖	1.17	1.47
Vaccenic acid	Fatty acids and conjugates	1.27	0.27	4.63‖
Caprylic acid	Fatty Acyls	1.46§	0.85	1.71§
Myristic acid	Fatty Acyls	1.81§	1.46	1.24‖
Nonanoic acid	Fatty Acyls	1.68§	0.94	1.78§
Heptadecanoic acid	Fatty Acyls	1.82‡	1.05	1.73¶
Methyl hexadecanoate	Fatty Acyls	0.59#	0.75	0.79‖
Palmitoleic acid	Fatty Acyls	1.6¶	1.23	1.3
Cetyl alcohol	Fatty alcohols	2.16§	1.33§	1.62§
Glycolic acid	Hydroxy acids and derivatives	1.24‡	1.04	1.19‡
Lactic acid	Hydroxy acids and derivatives	1.08*	1	1.08*
β-Indole-3-acetic acid	Indolyl carboxylic acids and derivatives	2.2	0.97	2.27‖
Linoleic acid	Lineolic acids and derivatives	1.81†	0.96	1.88¶
1,5-Anhydrohexitol	Organooxygen compounds	0.68*	0.96	0.7*
Pyranose(tagatose/sorbose)	Organooxygen compounds	2.21**	1.07	2.07#
Threitol	Organooxygen compounds	0.94	1.16*	0.8†
Arabitol	Organooxygen compounds	0.95	1.12	0.84*
Lactose	Organooxygen compounds	0.67	1.02	0.66*
P-cresol	Phenols	0.77	0.52‖	1.48

Data are fold change. Statistical significance: *P < 0.05, †P< 0.01, ‡P< 0.001, §P< 0.000001 by Student's t-test. ‖P < 0.05, ¶P < 0.01, #P < 0.001, **P < 0.000001 by Mann-Whitney U test. All p-values are corrected by FDR (Benjamini-Hochberg procedure). Abbreviations: HMDB – Human Metabolome Database, NGT - Normal Glucose Tolerance, aGT–GDM – group with diagnosed GDM based on abnormal OGTT, aFPG–GDM– group with diagnosed GDM based on abnormal fast plasma glucose level.

Supplementary Table S3. ROC curve analyses.

	AUC (95% CI)		Sensitivity		Specificity	
Name	1	2	1	2	1	2
α-Hydroxybutyric acid	0.797 (0.692-0.879)‖	0.745 (0.654-0.823)‖	69.2	70.5	83.6	72.1
β-Hydroxybutyric acid	0.708 (0.596-0.805)‡	0.693 (0.599-0.777)§	92.3	61.4	50.8	73.5
Capric acid*<br>Nonanoic acid†	0.716 (0.605-0.812)‡	0.669 (0.574-0.755)‡	69.2	68.2	79.1	63.2
Lauric acid	0.736 (0.625-0.828)‡	0.677 (0.582-0.762)§	69.2	72.7	76.1	60.3
Myristic acid	0.759 (0.650-0.848)§	0.787 (0.700-0.859)‖	76.9	77.3	73.1	73.5
Palmitic acid	0.745 (0.635-0.836)‡	0.754 (0.663-0.830)‖	84.6	93.2	70.2	45.6
Oleic acid	0.705 (0.592-0.802)‡	0.682 (0.587-0.767)§	92.3	84.1	55.2	45.6
FA	0.747 (0.638-0.838)§	0.775 (0.686-0.848)‖	92.3	59.1	52.2	83.8
α-Hydroxybutyric acid<br>β-Hydroxybutyric acid<br>FA	0.772 (0.664-0.858)	0.815 (0.730-0.882)	92.3	86.4	58.2	63.2
α-Hydroxybutyric acid<br>β-Hydroxybutyric acid<br>Myristic acid	0.791 (0.686-0.874)‖	0.828 (0.745-0.892)‖	84.6	72.7	68.7	79.4

1trimester, †2trimester. Statistical significance; ‡P < 0.01, §P < 0.001, ‖P < 0.0001. FA: Capric acid/Nonanoic acid†, Lauric acid, Myristic acid, Palmitic acid, Oleic acid. Abbreviations: AUC – Area under the curve, CI – Confidence Interval, FA - Fatty Acids

Supplementary Figure S1. Multivariate analysis results of fingerpritnting data.

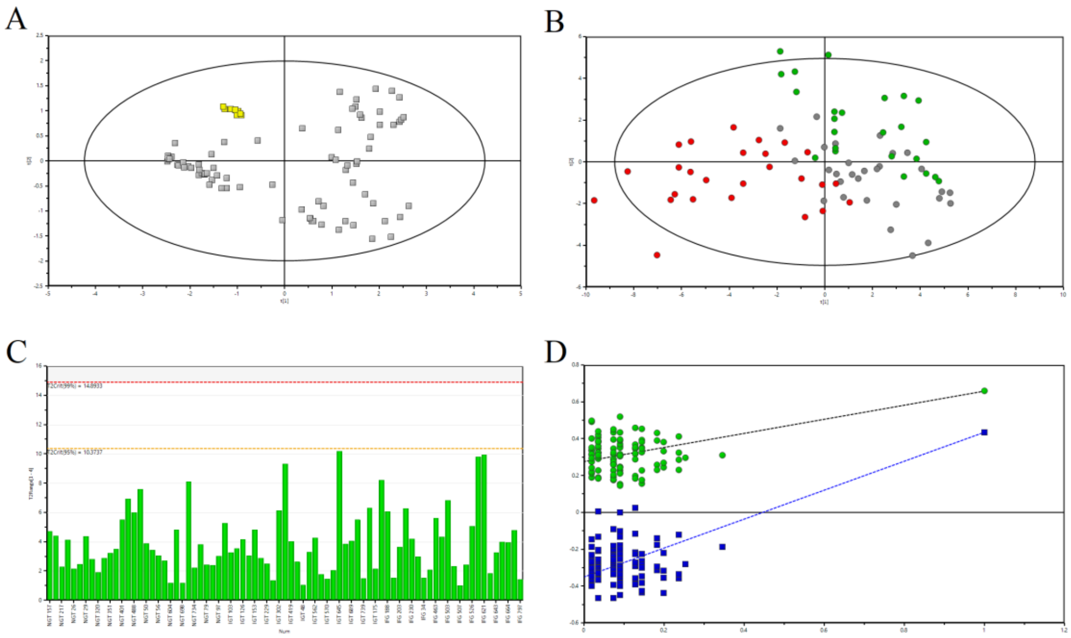

(A) Principal Component Analysis (PCA) plot generated using all samples (■) and QC samples (■). (R^2_{cum}=0.858, Q^2_{cum}=0.644, CTR).

(B) Partial Least Squares-Discriminant Analysis (PLS-DA) plot generated from the comparison between aGT-GDM (●), aFPG-GDM (●), and NGT (●) patients. (R^2_{cum}=0.549, Q^2_{cum}=0.506, UV scaling).

(C) Hotelling's Column Plot – displays the distance from the origin in the model plane for each selected observation. All the observations lie in the Hotelling's T 95% confidence range, indicating that there were no outliers.

(D) Permutation Plot. The validation plot generated from 100 permutation tests, demonstrated that the PLS-DA model was not random and overfitting as both permuted R^2 (●) and Q^2 (■) values were significantly lower than the corresponding original values and the blue regression line of Q^2 points had a negative intercept).

Abbreviations: R^2 – coefficient for variance explained, Q^2 – coefficient for variance predicted, UV – Unit Variance, CTR – centering, NGT - Normal Glucose Tolerance, aGT–GDM – group with diagnosed GDM based on abnormal OGTT, aFPG–GDM– group with abnormal fast plasma glucose.
